# Comparison of Preconception Diet Scores Across Studies: The PrePARED Consortium

**DOI:** 10.3390/nu17122035

**Published:** 2025-06-18

**Authors:** Lixuan Ji, Janaki Sundaresan, Cailey Cranny, Ke Pan, Danielle Symons Downs, Erica P. Gunderson, Gita Mishra, Abigail Pauley, Kaitlin S. Potts, James M. Shikany, Daniela Sotres-Alvarez, Lauren A. Wise, Emily W. Harville

**Affiliations:** 1 School of Medicine, Tulane University, New Orleans, LA 70112, USA; lji1@tulane.edu; 2 Department of Epidemiology, Celia Scott Weatherhead Tulane School of Public Health and Tropical Medicine, New Orleans, LA 70112, USA; jsundaresan@tulane.edu (J.S.); kpan@tulane.edu (K.P.); kspotts@bwh.harvard.edu (K.S.P.); 3 Department of Kinesiology, College of Health and Human Development, Pennsylvania State University, University Park, PA 16802, USA; dsd11@psu.edu (D.S.D.); amp34@psu.edu (A.P.); 4 Division of Research, Kaiser Permanente Northern California, Oakland, CA 94612, USA; erica.gunderson@kp.org; 5 School of Public Health, University of Queensland, Herston, QLD 4006, Australia; g.mishra@sph.uq.edu.au; 6 Division of Sleep Medicine, Harvard Medical School, Boston, MA 02115, USA; 7 Division of Preventive Medicine, University of Alabama-Birmingham, Birmingham, AL 35294, USA; jshikany@uabmc.edu; 8 Department of Biostatistics, University of North Carolina-Chapel Hill, Chapel Hill, NC 27516, USA; dsotres@email.unc.edu; 9 Department of Epidemiology, Boston University School of Public Health, Boston, MA 02118, USA; lawise@bu.edu

**Keywords:** nutrition, diet, data harmonization, preconception, dietary guidelines

## Abstract

Background: Preconception diet and nutritional status are important determinants of reproductive and pregnancy health. As a comprehensive evaluation, this paper describes harmonization of diet data across multiple cohorts including over 50,000 participants and the differences between them. This information may be useful for developing targeted strategies to improve women’s diet prior to pregnancy for optimal prenatal health outcomes. Methods: The Preconception Period Analysis of Risks and Exposures influencing health and Development (PrePARED) consortium incorporates studies covering the preconception period and includes both couples planning pregnancy and studies covering the reproductive period but not focused on pregnancy. We harmonized data on 56,520 participants from seven cohort studies that collected data during the preconception period. We generated data on diet quality according to the International Federation of Gynecology and Obstetrics (FIGO) nutrition checklist to examine diet quality measures across the cohorts and compare estimates of diet quality across studies. Four studies used food frequency questionnaires; one used a study-specific diet history; one used two 24 h dietary recalls; and one used a short series of general diet questions. Positive responses on the six FIGO questions were tallied to calculate a total diet quality score. Results: Cohort samples varied in terms of age; socioeconomic status; race; ethnicity; and geographic region. Across the cohorts, participants met a median of three or four of the FIGO criteria for diet quality; those most commonly met were recommendations for consumption of meat and protein, while those least commonly met were recommendations for limiting consumption of processed foods and snacks. There was greater variation in meeting recommendations for the consumption of fruits and vegetables; dairy; fish; and whole grains. The percentage meeting ≤ 2 criteria ranged from 6.4% (Coronary Artery Risk Development in Young Adults) to 40.4% (Bogalusa Heart Study). Discussion: There was wide variability across preconception cohort studies in the extent to which participants met FIGO dietary guidelines. Although studies were conducted in populations that were not likely to be malnourished, it was rare for women to meet all the preconception dietary recommendations. These findings illustrate a need for strategies to promote meeting dietary guidelines prior to conception to improve health outcomes.

## 1. Introduction

Preconception diet and nutritional status are important determinants of reproductive and pregnancy health. Maternal pre-pregnancy nutritional status (often represented with body mass index [BMI]) is an important predictor of pregnancy health, as sufficient nutrition is necessary for conception and carrying to term. More recently, pre-pregnancy obesity, which can be an indicator of overconsumption or an unbalanced diet, has emerged as an issue of worldwide concern [1,2]. Although preconception micronutrient supplementation has been investigated [3,4], general preconception dietary recommendations and patterns have been less considered. A multi-site study of preconception nutritional improvement in the United Kingdom, Singapore, and New Zealand identified both site-specific and pooled dietary patterns, with one featuring vegetables/fruits/nuts (healthy), one fried potatoes/processed meat/sweetened beverages (less healthy), and one fish/poultry/noodles/rice (mixed). Greater adherence to the healthy pattern was generally associated with better health indicators but higher BMI. The components of healthy and less healthy patterns were consistent across sites, while the mixed pattern had more variation by country [5]. A Japanese study found that an overall general indicator of diet, the Balanced Diet Score, was one of the healthy preconception lifestyle factors associated with better pregnancy outcomes, but dichotomized into top 60%/low 40%, so it is difficult to compare dietary quality to other populations [6].

Habitual diet is notoriously difficult to assess. The large amount of measurement error caused by the day-to-day variation and the limitations of self-report of dietary intake mean that the error-to-signal ratio is high. Major options for measuring usual diet include food frequency questionnaires (FFQs) and 24 h dietary recalls (24HDRs), as well as questionnaires that address specific food groups, nutrients, eating behaviors, dietary patterns, or cultural practices. Consortia investigating the health effects of diet therefore must deal with complex data and measurement error when trying to harmonize measures to improve power to address rarer outcomes. The ALPHABET consortium of European pregnancy cohorts derived a Dietary Approaches to Stop Hypertension (DASH) diet score [7] and an anti-inflammatory score [8] and noted wide between-cohort variability. The NutriGen alliance of four Canadian birth cohorts harmonized FFQs and dietary patterns [9] and identified differences in the effect of these dietary patterns by ethnicity, suggesting that diverse population studies are necessary to fully understand optimal diet [10].

The Preconception Period Analysis of Risks and Exposures influencing health and Development (PrePARED) consortium addresses preconception health across epidemiologic cohorts that cover the pre-pregnancy period. The consortium includes studies of couples actively planning pregnancy and others that evaluate health during the reproductive period but captured successive preconception periods and their pregnancy outcomes and thus characterize the natural history of childbearing. While a large portion of pregnancies are unplanned, those planning pregnancy often differ in their health behaviors [11,12]. This variation thus provides better generalizability but brings additional complications in terms of timing of measurement and sample inclusion criteria. This analysis is part of a larger effort to harmonize diet data in the consortium, addressing several distinct dietary patterns and recommendations, with the ultimate goal of a comprehensive evaluation of preconception diet and pregnancy and reproductive outcomes. In this paper, we present the methods of harmonization of dietary data and compare results across several cohorts with respect to a simple pregnancy-related dietary assessment, the International Federation of Gynecology and Obstetrics (FIGO) nutrition checklist [13].

## 2. Materials and Methods

### 2.1. Study Population

The PrePARED consortium incorporates studies covering the preconception period [14] and includes both couples planning pregnancy and studies covering the reproductive period but not specifically focused on pregnancy (referred to here as general-population studies for brevity). A description of the harmonization methods has been provided elsewhere [15]. This analysis included participating cohorts with dietary assessment. Details of dietary measures and validation where available are provided in Table S1, with a simplified flowchart in Figure S1. For each cohort, the overall study sample (all women of reproductive age) and the subpopulation that had at least one birth after baseline were examined. Although the dietary experience of all women of reproductive age, regardless of pregnancy intention, is of interest when assessing preconception health, for some of the cohorts (California Teachers Study [CTS], Hispanic Community Health Study/Study of Latinos [HCHS/SOL]) the overall sample had a large proportion of women at the older end of their reproductive years, whose childbearing likely occurred earlier in life.

The Australian Longitudinal Study of Women’s Health (ALSWH) is a longitudinal survey recruiting a nationally representative sample [16]. Data from the 1973–1978 cohort was used for this project (participants aged 18–23 years at baseline), and 14,247 women were enrolled. ALSWH assesses women’s physical and mental health, psychosocial aspects of health, and use of health services. Eight surveys were completed in the 1973-78 cohort from 1996 to 2018, and diet data were collected at survey 3 (2003–2004) and survey 5 (2009–2010) using the Dietary Questionnaire for Epidemiologic Studies, an FFQ [17].

The Bogalusa Heart Study (BHS) is a study of early-life predictors of cardiovascular disease in a semirural population in Louisiana, USA (65% White, 35% Black), having started in 1973, with participants currently in midlife [18]. Initially, data were collected in 23 cross-sectional surveys among children aged 4–17 years and adults aged 18–50 years, with subsequent longitudinal follow-up. The Bogalusa Babies study interviewed women about their reproductive history and linked it to vital statistics and birth records, where available. Dietary data were collected with the Youth-Adolescent Questionnaire, an FFQ designed for older children and adolescents, at the 2010–2013 exam [19,20]. Foods were matched to the Food Patterns Equivalent Database to identify food groups, including for foods that mapped to multiple food groups (such as sandwiches or tacos) [21].

Coronary Artery Risk Development in Young Adults (CARDIA) is a multicenter community-based longitudinal study examining the development and determinants of cardiovascular disease in young adults [22], having begun in 1985, in 5115 Black and White men and women aged 18–30 years in Birmingham, AL; Chicago, IL; Minneapolis, MN; and Oakland, CA. Women were not known to be pregnant when they attended the study exams. Characteristics for each pregnancy including gestational age, perinatal outcomes, dates of deliveries, and other characteristics were reported by women at the study baseline and at each follow-up in-person exam for all pregnancies between exams held every 2 to 5 years. Diet data were collected at baseline (1985–1986) and again at follow-up exams in year 7 (1992–1993) and year 20 (2005–2006) using a comprehensive diet history developed for the study [23].

The Central Pennsylvania Women’s Health Study (CePAWHS) [24] included a population-based survey of reproductive-aged women (18–45 years) in a 28-county target region of Central Pennsylvania. Women were recruited by random-digit dialing from September 2004 to March 2006, of which 1325 women were included who were able to become pregnant, both parous and nulliparous; 692 attended the baseline risk assessment, of which 362 completed the follow-up assessments. Nutritional data were collected through a set of six questions about usual diet.

The CTS is an observational cohort study, which started in 1995–1996, of 133,477 women who were members of the California State Teachers’ Retirement System [25]. This analysis was limited to those below age 45 years at baseline and with at least one follow-up. Five follow-ups have been conducted, most recently in 2019. Pregnancy data were collected at several follow-ups. Diet data was collected in at baseline, using the Block FFQ [26].

The HCHS/SOL is a multicenter population-based cohort study including 9835 self-identified Hispanic/Latino women aged 18–74 years, enrolled from 2008 to 2011 from four US communities (Bronx, NY; Chicago, IL; Miami, FL; and San Diego, CA) using a stratified multiple-stage area probability sample [27,28]. At the second clinic visit (2014–2017), a detailed pregnancy history questionnaire was collected for all pregnancies that lasted 6 months or longer after baseline. This analysis does not account for the complex survey design, and therefore findings describe only the convenience sample. Diet data were collected at baseline using two 24HDRs [29].

The Pregnancy Study Online (PRESTO) is a prospective cohort study of couples in the U.S. and Canada that began in 2013 [30]. Eligible primary participants were assigned female at birth, aged 21–45 years, and planning a pregnancy without the use of fertility treatment. After completing baseline questionnaires, participants are contacted by email every 8 weeks for up to 12 months or until self-reported pregnancy. Participants who conceive during follow-up are invited to complete questionnaires during pregnancy and postpartum. Diet data are collected 30 days after cohort entry, using the NCI’s web-based Diet History Questionnaire (DHQ II: 2013–2019 or III: 2020–present), an FFQ [30]. About 63% of the enrolled cohort completed the DHQ (II: 63.8%, III: 36.6%).

### 2.2. Harmonization Process

For harmonization, we followed the crosswalk–categorization–harmonization process outlined in our previous publications [15]. In this case, the diet domain was mapped to the diet variables across studies; in most cases, this was trivial because a separate dietary instrument was used (Table S1). Given the complexity of diet data, identifying common data elements, normally the next step, was not possible. In order to standardize time frames to the extent possible, if a study had repeated measures of diet, the one closest in time but prior to the first post-baseline pregnancy was used; for participants without pregnancies, the first measure was used. We used each study’s diet data to assess adherence to the FIGO recommendations. Most cohorts had previously conducted dietary analyses and provided calculated data for servings of meat/chicken, fruits/vegetables, fish, dairy, and whole grains, or provided information on subgroups within these categories (such as fruits and vegetables separately), which were then summed. The exceptions were ALSWH and HCHS, which only had reported servings of individual foods and thus required summing across all foods. Whole-grain carbohydrates were calculated based on servings of whole-grain foods, most frequently cereals and whole-grain breads. The category of “packaged snacks, cakes, pastries, or sugar-sweetened drinks” was calculated for each study and varied by the foods included, but usually included chips, sweets, sugar-sweetened beverages, and ice cream. If servings were not already defined, we used USDA guidelines (i.e., one piece or cup of fruits or vegetables as a serving, 3 oz. or 100 g of meat or fish, 1 cup of milk) [31]. Information from the dietary measure in each cohort was converted to servings per day or week and then compared to the FIGO criteria (see Table S3 for details of included foods by study).

### 2.3. Diet Recommendations

We chose the FIGO nutrition checklist as a simple instrument to examine dietary intake across the cohorts and assess baseline possibilities for harmonization. It is designed for women during preconception and early pregnancy and is a short checklist that assesses diet quality in clinical settings [13,32,33]. The “quality of diet” portion of the FIGO nutrition checklist consists of six dietary intake questions presented as a short FFQ (Table S2), incorporating number of times per week (or day) of intake of certain food categories. We used these questions to calculate the FIGO diet quality score, as suggested by Tsoi and associates [34]. For each question, participants with a number of servings that corresponded to a positive response received one point. We then summed all points to obtain a total diet quality score. The score ranged from 0 to 6, with higher scores indicative of better diet quality. We analyzed this score as a categorical variable.

### 2.4. Assessment of Covariates

Seven demographic variables were examined: (1) year at baseline for each cohort study, (2) participants’ age at baseline, (3) participants’ age at first pregnancy, (4) level of education, (5) race and ethnicity, (6) smoking status, and (7) level of alcohol intake. Details of the categories are provided in the tables. Relevant clinical factors examined were BMI at baseline or before first pregnancy and pre-existing hypertension and diabetes; depending on the cohort, these may have been identified by research biochemical laboratory tests, self-reported, measured at exams by study staff or based on treatment with medication.

### 2.5. Statistical Analysis

We performed all data management and statistical procedures using SAS version 9.4 (SAS Institute, Inc., Cary, NC, USA). Frequencies and descriptive statistics for categorical variables (e.g., education level, race/ethnicity) and continuous variables (age, BMI) were expressed as *n* (%) and as mean ± standard derivation (SD), respectively. Adherence to individual FIGO categories and overall score (sum of total recommendations met, 0–6) were calculated and a chi-square (χ^2^) test performed to examine statistical differences across cohorts, in the overall samples and in those with pregnancies. Radar charts were created using Microsoft Excel.

The original studies were approved by the institutional review boards of their relevant institutions and the combined data analysis has been approved by the Tulane University Institutional Review Board.

## 3. Results

Cohorts varied widely in demographic and other factors (Table 1). For instance, 80% of PRESTO participants and 99% of CTS participants had a college degree, while only around one third of the BHS, HCHS/SOL, and CePAWHS participants did. One third of BHS and half of the CARDIA participants (by design) self-identified as Black, but fewer than 5% of participants from the other cohorts did. By design, 100% of HCHS/SOL participants were Hispanic/Latina, whereas all other cohorts had only a small proportion in that category. These patterns were similar for the pregnancy cohorts (Table 2).

In each cohort, the median overall FIGO score (Table 3 and Table 4), was either 3 or 4. The percentage of participants meeting five or more criteria in each cohort ranged from 13.2% (BHS) to 40.4% (ALSWH).

**Figure 1:**
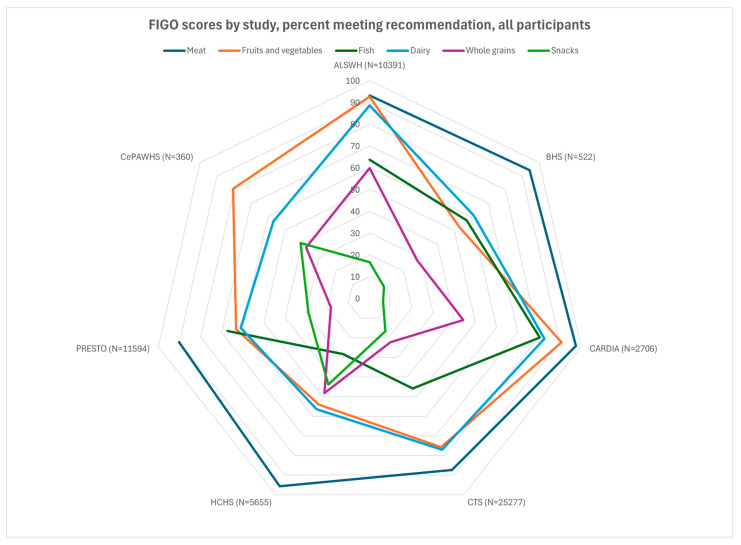
FIGO scores by study, percent meeting recommendation, all participants. Missing data on *n* = ALSWH, 1; BHS, 11; CARDIA, 2; Recommendation: meat/chicken 2–3 times/week, fruits/vegetables 2–3 times/day; fish 1–2 times/week; dairy every day; whole grains daily; packaged snacks, cakes, pastries, or sugar-sweetened drinks < 5 times/week. ALSWH, Australian Longitudinal Study of Women’s Health; BHS, Bogalusa Heart Study; CARDIA, Coronary Artery Risk Development in Young Adults; CePAWHS, Central Pennsylvania Women’s Health Study; CTS, California Teachers’ Study; FIGO, Federation Internationale de Gynecologie et d’Obstetrique (International Federation of Gynecology and Obstetrics); HCHS/SOL, Hispanic Community Health Study/Study of Latinos; PRESTO, Pregnancy Study Online.

**Figure 2:**
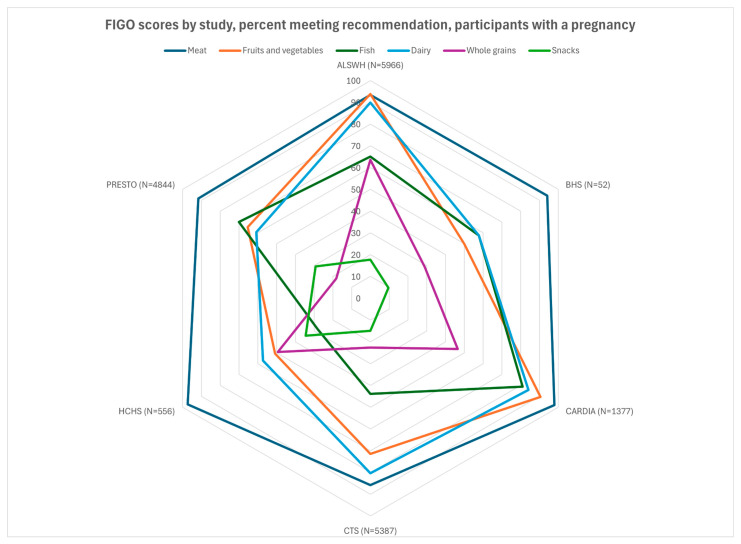
FIGO scores by study, percent meeting recommendation, participants with a pregnancy. BHS, missing data on *n* = 2.

Participants from all cohorts resided in Western countries, and a large majority in all cohorts met the criterion for meat consumption (2–3 times/week, Figure 1 and Figure 2). A majority did not meet the criteria for snacks and packaged foods. There was more variation for the other factors. The fruit and vegetable criterion was met by almost all participants in ALSWH (93%) and a large majority (>75%) of CARDIA, PRESTO, and CePAWHS participants but by fewer participants in BHS and HCHS/SOL (50–55%). The dairy criterion was met for almost all ALSWH participants but lower proportions of BHS, HCHS/SOL, PRESTO, and CePAWHS women (56–61%). The percentage of participants adhering to recommendations for fish consumption ranged from 28% (HCHS) to 67% (PRESTO). Adherence to recommendations for consumption of whole grains varied but was in the middle 50% for almost all cohorts (range: 22% for CTS to 64% for ALSWH).

## 4. Discussion

In this analysis, the extent to which participants adhered to dietary recommendations varied by cohort within a consortium of multiple preconception studies. Across the studies, however, it was rare for women to meet all the dietary recommendations, in particular the one addressing packaged snacks and sweets. In many of the studies a significant percentage of participants (~30%) were meeting ≤ 2 of the recommendations, suggesting that attempting to determine the ideal dietary pattern may be somewhat irrelevant when even basic dietary recommendations are not being met. Generally, the distribution of scores was similar between the overall and the pregnancy sample. Because we wished to characterize overall dietary patterns rather than examining predictors of fertility (a project for future analyses that will require a more extensive characterization of the population at risk for pregnancy), we did not formally examine differences between them. In other studies addressing general pre-pregnancy diet, a UK study of women planning pregnancy found that about half consumed five servings of fruits and vegetables more than 4 days/week, comparable to some of our studies but lower than others [35], while British birth cohorts across multiple generations suggested low intake of fruit preconception [36]. The European ALPHABET consortium found that the large majority consumed at least one serving of dairy per day, consistent with this analysis [7].

Some of these cohorts have examined pre-pregnancy diet within their individual studies. ALSWH examined preconception and pregnancy diet relative to the Australian Recommended Food Score and found that preconception women met roughly 40% of the recommendations on average for its subcomponents (vegetables, fruits, grains, protein, dairy, fat), with scores particularly low on protein (especially nuts/beans/soya) and whole grains [37]. The more general cohort (who did not necessarily have a later pregnancy) showed even lower adherence to the dietary recommendations [38]. HCHS/SOL noted generally poor pre-pregnancy diet quality [39], with average Healthy Eating Index (HEI) scores below 50. PRESTO examined diet relative to subfertility and spontaneous abortion, generally finding little association with iron [40] and dairy [41]; modest positive associations with high glycemic load, added sugar [42], trans fatty acids, and inflammatory diets [43]; and inverse associations with diets high in folate [44] and omega-3 fatty acids [45]. In CePAWHS, vegetable consumption was associated with higher birthweight [46]. CARDIA examined dietary changes induced by pregnancy and generally found increased energy, fat, and fiber intakes after pregnancy, with decreased fast food intake [47]. In addition, pre-pregnancy animal protein intake was associated with a higher risk of gestational diabetes mellitus [48]. The other cohorts did not analyze their data as preconception, although BHS characterized dietary changes over time [49,50] and associations of dietary patterns with cardiometabolic outcomes [51]. CTS analyses have largely been limited to associations with cancer [52,53,54].

### Strengths and Limitations

Strengths of the analysis include the prospective dietary data collection relative to pregnancy; while several studies of preconception diet have collected pre-pregnancy data retrospectively [55,56,57], dietary measures collected during pregnancy are likely to be affected by the dietary changes that often occur during this time (due to nausea, food aversions, increased appetite, etc.) and increased social desirability bias. Limitations include the difficulty of comparing studies with different populations, study designs, and diet measurement tools, as well as the inherent limitations of assessing dietary intake through questionnaire data. Measurement affected the distribution of diet quality, most notably in the CePAWHS study, where only a few questions assessed diet and did not address the entire FIGO checklist. HCHS/SOL was the only study to use 24HDRs instead of an FFQ or diet history, which may have led to its relatively low proportion of women reporting they ate packaged snacks (because any food not eaten daily might not be eaten in a 24 hr window). This also limits our ability to compare how results might differ due to the dietary measure used, i.e., FFQ vs. 24 h recall vs. the general questions used in CePAWHS. The FIGO checklist is designed as a general nutritional checklist that can be used internationally, and some diets that have generally been shown to be nutritionally adequate, such as vegetarian diets [58], would not meet the criteria. Some studies even indicate that higher preconception protein intake and meat and dairy intake are associated with gestational diabetes [48,59,60,61,62], and plant-based diets have been associated with a lower risk of hypertensive disorders of pregnancy [63]. However, participants were far more likely not to meet the packaged snack or whole-grain criteria, rather than the meat or fish criteria. This analysis is limited to consortium studies of participants who are largely based in the United States, Australia, or Canada, and so does not represent the global (non-Western) population or many nutritional patterns in and outside these countries; for instance, a study of Bedouin Arabs in Israel found substantially lower intakes of meat and poultry [64].

## 5. Conclusions

In this analysis, we demonstrate overall variation in nutritional status across preconception cohorts, generally sufficient intake of meat across a diverse set of preconception cohorts, and a high consumption of packaged snacks and sweets. Overall, we observed wide variability across preconception cohort studies in the extent to which participants met FIGO dietary guidelines. Although studies were conducted in populations that to our knowledge were not malnourished, the majority of women met all of the preconception dietary recommendations, suggesting there is a need for research and practical strategies to promote comprehensive nutritional improvements prior to conception, as this may set the stage for better maternal and infant health outcomes. Future research should also consider examining associations between dietary measures and reproductive and pregnancy outcomes to better understand the extent to which nutrition status mitigates adverse effects of obesity on pregnancy outcomes. Our consortium plans future analysis to identify the most important predictors of preconception diet, analyses of more detailed dietary recommendations such as the Mediterranean diet and Healthy Eating Index, and examining the associations of these preconception dietary factors with fertility, maternal health, and pregnancy outcomes.

## Figures and Tables

**Table 1:** Demographic, behavior, and BMI characteristics by cohort.

	ALSWH (1973–1978) (*n* = 10,392)	BHS (Women Aged ≤ 45 at Baseline) (*n* = 533)	CARDIA (*n* = 2709)	CePAWHS (*n* = 360)	CTS (*n* = 25,277)	HCHS/SOL (*n* = 5655)	PRESTO (*n* = 11,594)
**Age at baseline (years)**	20.78 ± 1.45	9.68 ± 3.12 21.96 ± 3.53 *	24.91 ± 3.69	25.97 ± 4.98	36.84 ± 5.57	37.42 ± 9.81	30.56 ± 4.09
**Education**							
Less than high school (%)	596 (5.7)	40 (7.5)	0 (0)	20 (5.6)	0/15,250 (0)	1739/5648 (30.8)	54 (0.5)
High school (%)	1010 (9.7)	165 (31.0)	757 (27.9)	105 (29.2)	2/15,250 (0.01)	1581/5648 (28.0)	451 (3.9)
Associates or some college (%)	3485 (33.5)	166 (31.2)	259 (9.6)	118 (32.8)	90/15,250 (0.59)	809/5648 (14.3)	2122 (18.3)
College and more (%)	5301 (51.0)	161 (30.3)	1693 (62.5)	117 (32.5)	15,158/15,250 (99.4)	1519/5648 (26.9)	8966 (77.3)
**Race/ethnicity**							
Hispanic/Latina (%)	**	0 (0.0)	0 (0)	11 (3.1)	1937/25,116 (7.7)	5655 (100)	756 (6.5)
White (%)		358 (67.3)	1284 (47.4)	330 (91.7)	20965/25,116 (83.5)	0 (0)	9729 (83.9)
Black or African American (%)		174 (32.7)	1425 (52.6)	11(3.1)	500/25,116 (2.0)	0 (0)	348 (3.0)
Asian, American Indian/Alaska Native, Native Hawaiian or Pacific Islander, mixed race, or other (%)		0 (0)	0 (0)	2 (0.8)	1714/25,116 (4.8)	0 (0)	1076 (2.2)
**Smoking**	5531 (53.2)	345 (64.7)	1417 (52.3)	112 (31.1)	4997/25,258 (19.8)	1565/5647 (28)	2310/11,591 (19.9)
**Alcohol use**	4070 (39.2)	125/486 (25.7)	1143/2708 (42.2)	33 (9.2)	6744 (26.7)	260/3969 (7)	1591/11,592 (13.7)
**BMI (pre-pregnancy or baseline visit)**	23.11 ± 4.57 (*n* = 10,176)	24.47 ± 5.63	24.5 ± 5.13 (*n* = 2707)	28.2 ± 7.52	24.12 ± 5.01 (*n* = 24,889)	29.91 ± 6.78	27.75 ± 7.40

* Age at first adult visit. ** Information on race was not collected in the Australian study in a way that it could be compared to the U.S.-based studies. ALSWH, Australian Longitudinal Study of Women’s Health; BHS, Bogalusa Heart Study; CARDIA, Coronary Artery Risk Development in Young Adults; CePAWHS, Central Pennsylvania Women’s Health Study; CTS, California Teachers’ Study; HCHS/SOL, Hispanic Community Health Study/Study of Latinos; PRESTO, Pregnancy Study Online.

**Table 2:** Demographic, behavior, and BMI characteristics by cohort: participants with pregnancy after diet measurements.

	ALSWH (1973–1978) (N = 5966)	BHS (N = 54)	CARDIA (N = 1381)	CTS (N = 5387)	HCHS/SOL (N = 556)	PRESTO (N = 4884)
	Mean ± SD or N(%)	Mean ± SD or N(%)	Mean ± SD or N(%)	Mean ± SD or N(%)	Mean ± SD or N(%)	Mean ± SD or N(%)
**Age at baseline**	20.7 ± 1.44	6.8 ± 1.37 23.4 ± 3.3	24.05 ± 3.67	31.45 ± 4.47	26.56 ± 5.92	30.08 ± 3.66
**Education**						
Less than high school (%)	217 (3.6)	2 (3.7)	0 (0)	0/2930 (0)	150 (27.1)	9 (0.2)
High school (%)	494 (8.3)	12 (22.2)	400 (29.0)	0/2930 (0)	183 (33.1)	97 (2.0)
Associates or some college (%)	1819 (30.5)	19 (35.2)	131 (9.7)	6/2930 (0.2)	55 (10.0)	686 (14.2)
College and more (%)	3436 (57.6)	21 (38.9)	850 (61.6)	2924/2930 (99.8)	165 (29.8)	4052 (83.7)
**Race/ethnicity**						
Hispanic/Latina (%)	**	0 (0)	0 (0)	508/5360 (9.5)	556 (100)	283 (5.8)
White (%)		36 (66.7)	681 (49.31)	4350/5360 (81.2)	0 (0)	4193 (86.6)
Black or African American (%)		18 (33.3)	700 (50.69)	54/5360 (1.0)	0 (0)	72 (1.5)
Asian, American Indian/Alaska Native, Native Hawaiian or Pacific Islander, mixed race, or other (%)		0 (0)	0 (0)	448/5360 (8.4)	0 (0)	296 (6.1)
**Age at first pregnancy after baseline**	30.56 ± 4.75	26.16 ± 8.17	28.20 ± 6.04 (*n* = 1295)	35.95 ± 4.76		30.2 ± 3.7
**Smoking**	3052 (51.2)	26 (48.2)	735 (53.2)	783/5385 (14.5)	127/555 (22.9)	806/4842 (16.7)
**Alcohol use**	2448 (41.0)	4 (7.7)	589/1380 (42.7)	1365 (25.3)	31/400 (7.8)	624 (12.9)
**BMI (pre-pregnancy)**	24.04 ± 4.7 (*n* = 5812)	26.42 ± 6.1	25.66 ± 5.87	23.27 ± 4.27	28.25 ± 6.46	26.46 ± 6.30

** Information on race was not collected in the Australian study in a way that it could be compared to the U.S.-based studies. ALSWH, Australian Longitudinal Study of Women’s Health; BHS, Bogalusa Heart Study; CARDIA, Coronary Artery Risk Development in Young Adults; CePAWHS, Central Pennsylvania Women’s Health Study; CTS, California Teachers’ Study; HCHS/SOL, Hispanic Community Health Study/Study of Latinos; PRESTO, Pregnancy Study Online.

**Table 3:** FIGO scores by study—all participants.

	ALSWH	BHS	CARDIA	CTS	HCHS/SOL	PRESTO	CePAWHS ^†^	*p*-Value
	*n* = 10,391 * (%)	*n* = 522 (%) ^§^	*n* = 2706(%) **	*n* = 25,277(%)	*n* = 5655 (%)	*n* = 11,514 (%)	*n* = 360 (%)	
0	7 (0.07)	11 (2.1)	0 (0)	123 (0.49)	10 (0.18)	80 (0.69)	30 (8.3)	<0.0001 *
1	65 (0.63)	62 (11.9)	22 (0.81)	1497 (5.9)	377 (6.7)	732 (6.3)	85 (23.6)	
2	478 (4.6)	110 (21.0)	173 (6.39)	4648 (18.4)	1142 (20.2)	2102 (18.1)	96 (26.7)	
3	1899 (18.3)	135 (25.8)	496 (18.3)	8184 (32.4)	1702 (30.1)	3377 (29.1)	97 (26.9)	
4	3848 (37.0)	136 (26.0)	1080 (39.3)	7648 (30.3)	1552 (27.4)	3651 (31.5)	52 (14.4)	
5	3613 (35.8)	69 (13.2)	904 (33.4)	2849 (11.3)	745 (13.2)	1470 (12.7)	0 (0)	
6	481 (4.6)	0 (0)	33 (1.2)	328 (1.3)	127 (2.3)	182 (1.6)	0 (0)	

ALSWH, Australian Longitudinal Study of Women’s Health; BHS, Bogalusa Heart Study; CARDIA, Coronary Artery Risk Development in Young Adults; CePAWHS, Central Pennsylvania Women’s Health Study; CTS, California Teachers’ Study; FIGO, Federation Internationale de Gynecologie et d’Obstetrique (International Federation of Gynecology and Obstetrics); HCHS/SOL, Hispanic Community Health Study/Study of Latinos; PRESTO, Pregnancy Study Online. Missing data on *n* = * 1, ** 2, ^§^ 11; ^†^ CePAWHS omitted from chi-square analysis.

**Table 4:** FIGO scores by study—pregnancies.

	ALSWH	BHS	CARDIA	CTS	HCHS	PRESTO	*p*-Value
	*n* = 5966 (%)	*n* = 52 * (%)	*n* = 1377 (%)	*n* = 5387 (%)	*n* = 553 (%)	*n* = 4844 (%)	
0	2 (0.03)	1 (1.9)	0 (0)	27 (0.5)	1 (0.18)	19 (0.4)	<0.0001
1	29 (0.49)	4 (7.7)	7 (0.51)	375 (7.0)	41 (7.4)	273 (5.6)	
2	233 (3.9)	16 (30.8)	81 (5.9)	1062 (19.7)	118 (21.2)	805 (16.6)	
3	1069 (17.9)	12 (23.1)	245 (17.8)	1708 (31.7)	179 (32.2)	1424 (29.4)	
4	2202 (36.9)	12 (23.1)	542 (39.4)	1577 (29.3)	146 (26.3)	1628 (33.6)	
5	2144 (35.9)	7 (13.5)	490 (35.6)	573 (10.6)	62 (11.2)	620 (12.8)	
6	287 (4.8)	0 (0)	12 (0.87)	65 (1.2)	9 (1.6)	75 (1.6)	

* Missing data on 2. ALSWH, Australian Longitudinal Study of Women’s Health; BHS, Bogalusa Heart Study; CARDIA, Coronary Artery Risk Development in Young Adults; CTS, California Teachers’ Study; FIGO, Federation Internationale de Gynecologie et d’Obstetrique (International Federation of Gynecology and Obstetrics); HCHS/SOL, Hispanic Community Health Study/Study of Latinos; PRESTO, Pregnancy Study Online.

## Data Availability

The data presented in this study are available on request from the corresponding author. Data are not publicly available due to protections on participant confidentiality.

## Supplementary Materials

The following supporting information can be downloaded at https://www.mdpi.com/article/10.3390/nu17122035/s1, Figure S1: Flowchart for the dietary analysis of the overall sample and birth group in the Preconception Period Analysis of Risks and Exposures Influencing health and Development (PrePARED) consortium (1973-present); Table S1: Diet data collection across cohorts; Table S2: FIGO dietary questionnaire; Table S3: Details of categories in the FIGO dietary measure. References [65,66,67,68,69,70,71,72,73,74] are cited in the supplementary materials.
