# Supplementary material for: Comparison of Preconception Diet Scores Across Studies: The PrePARED Consortium
Source: Nutrients. 2025 Jun 18;17(12):2035. doi: 10.3390/nu17122035 (PMC12196022; doi:10.3390/nu17122035)
Supplement: Supplementary file 1 [file nutrients-17-02035-s001.zip › nutrients-3626140-supplementary.pdf]

Table S1. Diet data collection across cohorts

Cohort	Dietary measure	Type	When measured	Details of measure	Validation
ALSWH	Dietary Questionnaire for Epidemiologic Studies	FFQ	Phase 3 and phase 5	74 foods, 6 alcoholic beverages, over last 12 months semiquantitative. Questions on the total intakes of fruit and vegetables are used to adjust the intakes of individual fruits and vegetables. Portion photographs of vegetables, potatoes, meat and casserole dishes are used to calculate a portion factor that is applied to scale up or down the standard portions of foods that showed variation by gender or ethnicity in the weighed food records from which the FFQ was derived.	Validated in middle-aged women;[65] associated with better self-rated health and lower health services usage in ALSWH[17]
BHS	Youth/Adolescent Questionnaire	FFQ	Multiple follow-ups	Semiquantitative, 151 items. Participants were older than youth/adolescent – instrument used to maintain comparability with earlier measures.	Compared with 3 24-hr recalls.[19] Associated with cardiovascular risk factors.[51]
CARDIA	CARDIA dietary history (developed specifically for CARDIA)	diet history	Baseline, year 7, year 20	Food intake over past month. 166 food groups, includes portion sizes. Converted to standard servings and nutrient data taken from Nutrition Data System for Research [66]	Correlated with expected nutrient intakes[23] and 24-hr diet recall[67]
CePAWHS	Study-specific	6 questions	Baseline	How often eat fruit, vegetables, green salad, snack foods, dairy, whole grains in a typical week	
CTS	Block dietary questionnaire	FFQ	Baseline	Frequency of consumption and portion size of 103 food and beverage items	Validated with 4 24-h recalls[26]
HCHS/SOL	24 h recall	Average of 2 24-hr recalls	24-h recall: Baseline and 5-90 d; first in-person, 2nd telephone	Multiple-pass methods of Nutrition Data System for Research software	Validated with nitrogen and doubly-labeled water[68]; associated with

					CVD[69] and metabolic syndrome[70]
PRESTO	Dietary Health Questionnaire (DHQ) II, a web-based food frequency questionnaire developed and validated by the National Cancer Institute	FFQ	Baseline	277 individual food items, average intake over last year	DHQ-1 validated by NCI relative to other checklists and 24-h recall[71-74]<br>No separate validation in this study. DHQ-II and III showed good-moderate reliability and validity.[73]

ALSWH, Australian Longitudinal Study of Women's Health; BHS, Bogalusa Heart Study; CARDIA, Coronary Artery Risk Development in Young Adults; CePAWHS, Central Pennsylvania Women's Health Study; CTS, California Teachers' Study; DHQ, dietary health questionnaire; FFQ, food frequency questionnaire; HCHS/SOL, Hispanic Community Health Study/Study of Latinos; PRESTO, Pregnancy Study Online

Table S2. FIGO dietary questionnaire[33]

i) Do you eat meat or chicken 2-3 times per week?
ii) Do you regularly eat more than 2 – 3 portions of fruit or vegetables per day?
iii) Do you eat fish at least 1-2 times per week?
iv) Do you consume dairy products (such as milk, cheese, yogurt) every day?
v) Do you eat whole grain carbohydrate foods (brown bread, brown pasta, brown rice or other) at least once a day?
vi) Do you consume packaged snacks, cakes, pastries or sugar-sweetened drinks less than 5 times a week?

FIGO, Federation Internationale de Gynecologie et d'Obstetrique (International Federation of Gynecology and Obstetrics)

Table S3. Details of categories in the FIGO dietary measure						
Cohort	Meat/chicken	Fruits and vegetables	Fish	Dairy	Whole grain	Packaged snacks, cakes, pastries, SSB
ALSWH	Bacon, beef, chicken, ham, lamb, pork, salami, sausages, veal, meat pies, hamburger	Apples, apricots, avocado, banana, broccoli, baked beans, bean sprouts, beets, cabbage, capsicum, carrots, cauliflower, celery, cucumber, fruit juice, garlic, green beans, lettuce, mango, melon, mushrooms, pineapple, pears, peas, peaches, onion, oranges, beans, spinach, pumpkin, tomato, canned fruit, strawberries, zucchini, tomato sauce	Fish, fried fish, canned fish	Butter, cream cheese, firm cheese, flavored milk, milk, hard cheese, cottage cheese, yogurt	High fiber breads, cereals	Cakes, chips (French fries), chocolate, crisps (potato chips), cookies, sugar, ice cream
BHS	Burgers, meat burritos, tacos, beef, beef stew, beef roast, pork, game, chicken, other poultry, liver, hot dog, ham, bacon, deli meat in sandwiches, meatballs, meatloaf, meat in pasta dishes, meat soups	Raisins, grapes, banana, cantaloupe or melons, apple or applesauce, pears, oranges, grapefruit, strawberries, peach, plums, apricots, orange juice, apple juice, tomatoes, string beans, broccoli, beets, corn, peas, lima beans, mixed vegetables, spinach, greens or kale, green or red peppers, yams or sweet potatoes, zucchini, summer squash, eggplant, carrots, celery, lettuce, coleslaw, vegetables in tacos, tomato-based pasta sauce, vegetables in egg rolls, ketchup, salsa	tuna salad, fish sticks, fish cakes, fish sandwich, shrimp, lobster, or scallops	Milk, breakfast drink, yogurt, cottage cheese, cheese, cheese on sandwich or in pasta dishes, cream soup	Cold and hot cereal, dark bread, popcorn, corn chips*, graham crackers*, crackers* (*foods were mapped to FPED database; these mapped partially to whole and partially to refined grains)	Soda, punch, sugared iced tea, potato chips, corn chips, nachos, popcorn, fruit rollups/fun fruit, crackers (saltines or Wheat Thins), Poptarts, cake, snack cakes, Danish or pastry, doughnuts, cookies, brownies, pie, chocolate, other candy bars, non-chocolate candy, Jello, pudding, frozen yogurt, ice

						cream, milkshake, popsicles
CARDIA	Meat (already coded): hamburgers, ground beef, casserole, pot roast, steak, ham, ham hocks, sausage, bacon, snack meats, cold cuts, cured pork, organ meats, fried chicken, game, poultry, fast-food chicken sandwiches, turkey, Cornish hen, duck, goose, turkey salad, lamb, veal	Fruit, vegetables (already coded): citrus fruit, dark green vegetables, yellow vegetables, fried vegetables, tomatoes, or lettuce salads, fresh, frozen, canned vegetables, pickles, olives	Fish (already coded): fresh, frozen, smoked, fast-food sandwiches, fish, shellfish, octopus, tuna salad, canned fish	Dairy (already coded); milk, yogurt, cream, butter, frozen desserts	Whole grains: loaf-type bread and plain rolls – whole grain; cakes, cookies, pies, pastries, Danish, doughnuts, and cobblers – whole grain; snack chips – whole grain; Crackers- whole grain; other breads (quick breads, corn muffins, tortillas) – whole grain; pasta – whole grain; ready-to-eat cereal (not presweetened) – whole grain; ready-to-eat cereal (presweetened); snack bars – whole grain; grains, flour and dry mixes – whole grains	Packaged (sugar, beverages): cake, crackers, chips, sweet tea, flavored milk, sweetened fruit juice, pretzels, popcorn, pie, toaster pastries, snack cakes, cream puffs, pudding, gelatin, cookies, brownies, ice cream, frozen dairy dessert, frozen nondairy dessert, pudding and other dairy dessert
CePAWHS	Not assessed	Fruit, green salad, vegetables not including carrots, potatoes, or salad	Not assessed	Milk, cheese, yogurt, not ice cream or frozen yogurt	Whole grains, including whole wheat bread, brown rice, cereal with fiber	Snack foods such as chips, cookies, ice cream, frozen yogurt, candy

CTS	Beef roast, liver, pork, sausage, bacon, hamburger, hotdogs, lunchmeat, chicken, turkey, chicken stew, fried chicken	Banana, apple, orange, grapefruit, cantaloupe, peaches, prune, strawberry, other fruit, watermelon, corn, tomatoes, broccoli, cauliflower, spinach, mustard greens, turnips, carrots, mixed vegetables, green salad, sweet potatoes, other vegetables	tuna, oyster, shellfish, other fish	Milk in cereal, cottage cheese, other cheese, frozen yogurt, whole milk, lowfat milk, skim milk, dairy creamer, milk in coffee, butter	Dark bread, fiber cereal, cooked cereal	Sweetened beverages, pumpkin pies, other pies, chocolate, candy, jelly, ice cream, muffins, bagels, buns, pancakes, granola bars, sweet cereals, cookies, cake, doughnuts
HCHS/SOL	beef, veal, lamb, pork, game, poultry, fried chicken, cold cuts and sausage, organ meats	citrus, non-citrus fruit, avocado and similar, dark-green vegetables, deep-yellow vegetables, tomato, other starchy vegetables, other vegetables	Fish, shellfish, including fried	milk, milk beverage powder, cheese, yogurt, cream, dairy-based meal replacements	Whole grain flour, whole grain bread and rolls, other whole grain breads, whole grain crackers, whole grain pasta, whole grain cereal, whole grain snack bars	Cakes, cookies, snack bars, chips, popcorn, sweetened water, sweetened fruit drinks, sweetened tea, frozen dairy dessert, pudding and dairy dessert, fruit-based savory snack, vegetable-based savory snack, candy, chocolate
PRESTO	bacon, beef mix in mixed dishes, chicken, chicken in mixed dishes, deli ham, roast beef in sandwiches, turkey or chicken cold	apple, asparagus, banana, broccoli, cabbage, cantaloupe, carrot, cauliflower, cooked greens, corn, dried fruit, fresh fruit, grape, grapefruit, green beans, melon, mixed vegetables, onion,	fish, fish sticks, fried shellfish, other fish, shellfish not fried, canned tuna	butter, cheese, chocolate milk, cottage cheese, frozen yogurt, and cheese, milk, yogurt	Oatmeal, grits or other cooked cereal, cold cereal, Total Raisin Bran, Total Cereal or Product 19, All Bran, Fiber One, 100% Bran or All-Bran Buds, other	Fruit drink, soda, soda, sports drink, Energy drink, energy drink, pancakes, crackers, potato chips, corn chips, frozen yogurt, ice cream, cake, cookies, doughnut,

	cuts, ground beef in mixtures, ground chicken or turkey, ham, hamburger from fast food, hamburger from not fast food, hot dogs, liver, pork, roast beef, sausage, spareribs, steak, turkey	orange, other vegetables, peach, pear, pineapples, raw greens, strawberry, tomato, yams, winter squash			bran or fiber cereal, any other type of cold cereal	muffin, fruit crisp, pie, chocolate candy, Other candy

ALSWH, Australian Longitudinal Study of Women's Health; BHS, Bogalusa Heart Study; CARDIA, Coronary Artery Risk Development in Young Adults; CePAWHS, Central Pennsylvania Women's Health Study; CTS, California Teachers' Study; FIGO, Federation Internationale de Gynecologie et d'Obstetrique (International Federation of Gynecology and Obstetrics); HCHS/SOL, Hispanic Community Health Study/Study of Latinos; PRESTO, Pregnancy Study Online

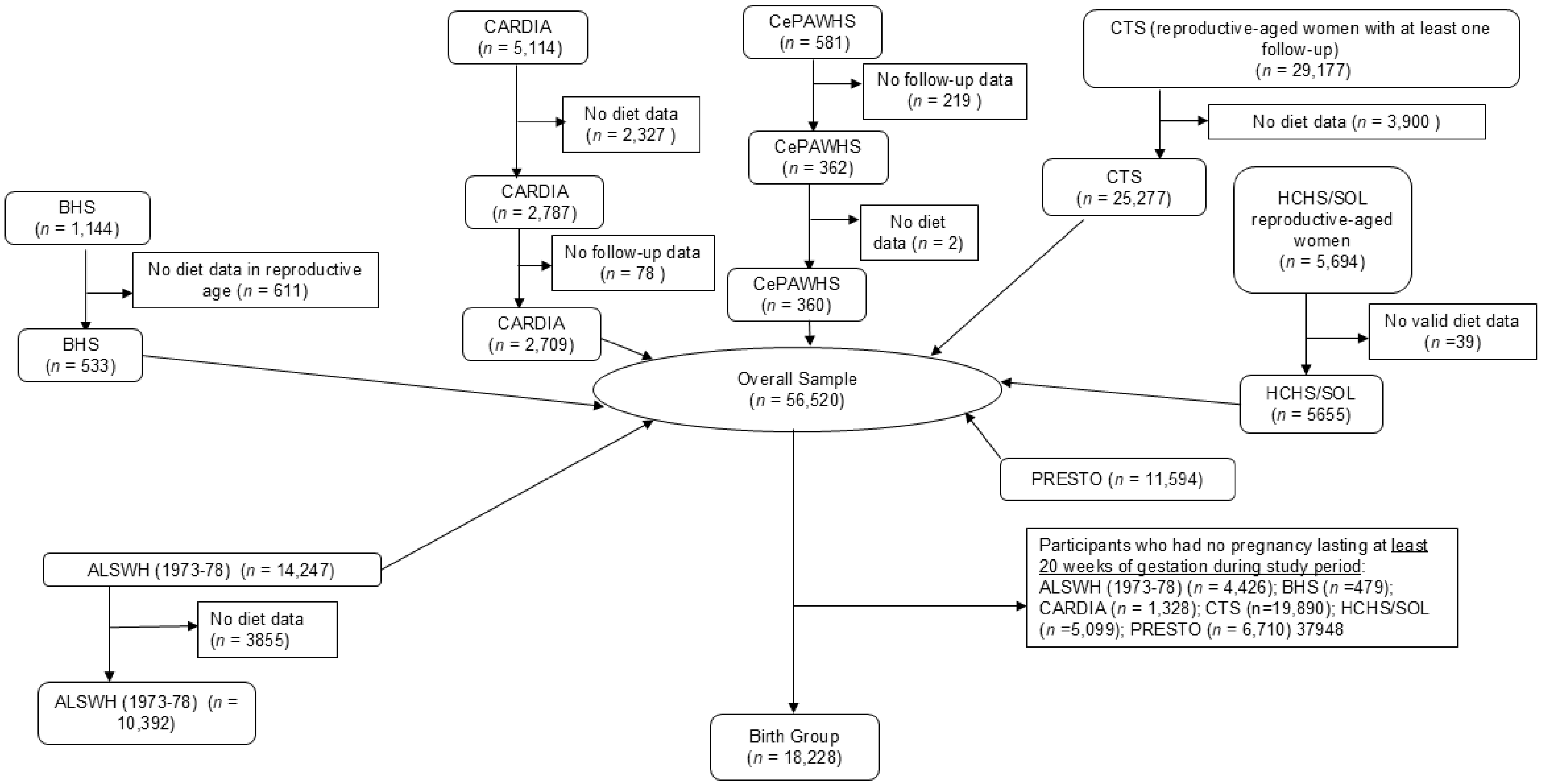

Figure S1. Flowchart for the dietary analysis of overall sample and birth group in the Preconception Period Analysis of Risks and Exposures Influencing health and Development (PrePARED) consortium (1973-present).

ALSWH: The Australian Longitudinal Study on Women's Health; BHS: Bogalusa Heart Study; CARDIA: Coronary Artery Risk Development in Young Adults; CePAWHS: Central Pennsylvania Women's Health Study; CTS, California Teachers' Study; HCHS/SOL: The Hispanic Community Health Study/Study of Latinos; PRESTO: Pregnancy Study Online. CePAWHS not included in birth group.

Figure S1.
